# Heritability of Psychotic Experiences in Adolescents and Interaction With Environmental Risk

**DOI:** 10.1001/jamapsychiatry.2022.1947

**Published:** 2022-08-03

**Authors:** Mark J. Taylor, Daniel Freeman, Sebastian Lundström, Henrik Larsson, Angelica Ronald

**Affiliations:** 1 Department of Medical Epidemiology and Biostatistics, Karolinska Institutet, Stockholm, Sweden; 2 Department of Psychiatry, University of Oxford, Oxford, United Kingdom; 3 Gillberg Neuropsychiatry Centre, Centre for Ethics, Law and Mental Health, University of Gothenburg, Gothenburg, Sweden; 4 School of Medical Sciences, Örebro University, Örebro, Sweden; 5 Genes Environment Lifespan Laboratory, Centre for Brain and Cognitive Development, Department of Psychological Sciences, Birkbeck University of London, London, United Kingdom

## Abstract

**Importance:**

Genetic risk factors are known to play a role in the etiology of psychotic experiences in the general population. Little is known about whether these risk factors interact with environmental risks for psychotic experiences.

**Objective:**

To assess etiological heterogeneity and exposure to environmental risks associated with psychotic experiences in adolescence using the twin design.

**Design, Setting, and Participants:**

This twin study, conducted from December 1, 2014, to August 31, 2020, included a UK-based sample of twin pairs aged 16 years. This investigation evaluated the extent to which the genetic variance underlying psychotic experiences and the magnitude of the heritability of psychotic experiences was moderated by exposure to 5 environmental risk factors (bullying, dependent life events, cannabis use, tobacco use, and low birth weight). Psychotic experiences were assessed by 5 self-reported measures and 1 parent-reported measure. Participants’ exposure to environmental risks was assessed at birth and age 12 to 16 years. Structural equation models were used to assess differences in the variance in and heritability of psychotic experiences across these exposures, while controlling for gene-environment correlation effects. Analyses were repeated in an independent Swedish sample. Data analyses were performed from September 1, 2018, to August 31, 2020.

**Main Outcomes and Measures:**

Primary outcome measures were exposure to environmental factors, as measured by a composite score, and psychotic experiences.

**Results:**

A total of 4855 twin pairs (1926 female same-sex pairs, 1397 male same-sex pairs, and 1532 opposite-sex pairs) were included from the Twins Early Development Study (TEDS), and 6435 twin pairs (2358 female same-sex pairs, 1861 male same-sex pairs, and 2216 opposite-sex pairs) were included from the Child and Adolescent Twin Study in Sweden (CATSS). Mean age of twins from TEDS was 16.5 years. Mean age of twins from CATSS was 18.6 years. More exposure to environmental risk factors was associated with having more psychotic experiences. The relative contribution of genetic influences to psychotic experiences was lower with increasing environmental exposure for paranoia (44%; 95% CI, 33%-53% to 38%; 95% CI, 14%-58%), cognitive disorganization (47%; 95% CI, 38%-51% to 32%; 95% CI, 11%-45%), grandiosity (41%; 95% CI, 29%-52% to 32%; 95% CI, 9%-48%), and anhedonia (49%; 95% CI, 42%-53% to 37%; 95% CI, 15%-54%). This pattern was replicated for the measure of psychotic experiences in the independent Swedish replication sample. The heritability of hallucinations and parent-rated negative symptoms remained relatively constant.

**Conclusions and Relevance:**

Findings of this twin study suggest that environmental factors play a greater role in the etiology of psychotic experiences than genetic factors. The relative importance of environmental factors is even higher among individuals exposed to environmental risks for psychotic experiences, highlighting the importance of a diathesis-stress or bioecological framework for understanding adolescent psychotic experiences.

## Key Points

**Question:**

Are psychotic experiences less heritable for adolescents who experience more environmental risk factors?

**Findings:**

In a twin study of 4855 twin pairs aged 16 years from the UK, the relative importance of genetic influences on certain psychotic experiences diminished with more exposure to environmental risk factors. A similar pattern of results was observed in an independent sample from Sweden (N = 8568 pairs).

**Meaning:**

These results suggest gene-by-environment interactions in relation to psychotic experiences; some groups of individuals with seemingly low genetic risk for psychotic experience may develop them if exposed to high levels of environmental risk, in a similar manner to clinical observations in relation to schizophrenia.

## Teaser

This twin study investigates whether the heritability of psychotic experiences changes with increasing exposure to environmental risks in adolescents.

## Introduction

Psychotic experiences, such as paranoia and hallucinations, are relatively common in adolescence,^1^ with an estimated prevalence of 5% in adults.^2^ Although they follow a transient course in some individuals, in others they are persistent and associated with increased risk for psychosis,^3^ psychiatric disorders,^4^ suicide,^5^ and physical disorders.^6^ Many associations with adverse outcomes are independent of psychiatric disorders,^6,7^ highlighting the need to understand psychotic experiences in their own right.

There has been considerable progress in understanding the etiology of psychotic experiences, including moderate twin heritability (15%-59%^8^) and single-nucleotide variation heritability of 3% to 9%.^9,10^ These estimates indicate that environmental factors play a considerable role in psychotic experiences. Factors found to be associated with psychotic experiences include bullying and childhood maltreatment,^11,12^ life events,^13,14^ cannabis use,^13,15^ and tobacco use.^16^ Early exposures, such as obstetric complications, have also been implicated.^16^ These exposures are heritable, however, and their association with psychotic experiences is partly attributable to genetic influences. For example, British twin studies found that the associations between psychotic experiences and bullying,^17^ life events,^18^ and tobacco use^19^ were explained by genetics, indicating that some of these associations are not causal.

This evidence suggests that genetic and environmental influences on psychotic experiences do not operate independently of one another. Another manner in which this may occur is through gene-by-environment interaction, whereby the importance of genetic influences may vary dependent on environmental exposure and vice versa. Two long-standing theoretical frameworks provide means to illustrate this. The diathesis-stress model posits that genetic susceptibility is required to trigger a response to environmental exposures. In the context of a twin study, this would lead to higher heritability estimates in communities with more environmental exposure compared with communities with less exposure.^20^ By contrast, the bioecological model posits that genetic factors become more pertinent in more favorable environments, leading to lower heritability in twin studies in the context of environmental exposure.^21^ It is unknown whether these models apply to psychotic experiences.

We aimed to test gene-by-environment interaction in relation to psychotic experiences using twin methods. We tested whether the genetic and environmental variance in these phenotypes varied as a function of exposure to environmental risks. After testing for these associations in a British sample of adolescents, we assessed whether our results could be replicated in a Swedish sample. We hypothesized that the genetic and environmental variance in psychotic experiences would fluctuate with exposure to environmental risks. We did not draw specific hypotheses about the direction of such changes owing to the paucity of existing evidence.

## Methods

### Study Populations

The Twins Early Development Study (TEDS) includes twins born in England and Wales from 1994 to 1996^22^ who participated in the Longitudinal Experiences and Perceptions Study (LEAP) at age 16 years. A total of 5059 of 10 874 invited families (47%) participated. The sample is representative of the UK population on various demographic characteristics, including ethnicity and socioeconomic status.^1^ We excluded participants with autism, genetic syndromes, chromosomal abnormalities, extremely severe obstetric complications, and those missing first-contact data. Zygosity was ascertained through DNA testing and a questionnaire assessing twin resemblance. TEDS has ethical approval from the King’s College London research ethics committee. TEDS participants provided written informed consent before participation.

The Child and Adolescent Twin Study in Sweden (CATSS) comprises families of Swedish twins who are invited to participate when the twins turn age 9 years.^23^ We used data collected from the participants at ages 15 and 18 years, which have respective response rates of 61% and 59%. CATSS is representative of the Swedish population on various characteristics.^24^ We excluded participants with chromosomal abnormalities or brain injuries. Zygosity was ascertained using a panel of single nucleotide variations or a questionnaire assessing twin similarity and reconfirmed for genotyped twins. CATSS has ethical approval from the Stockholm County Ethical Review Board. Informed consent was inferred from participant completion of questionnaires. The measures used in this study are described in eTable 1 in the Supplement.

#### Environmental Exposures

We selected 5 exposures, based on prior research: bullying, dependent life events, cannabis use, tobacco use, and low birth weight. We summed them to create an exposure score for each individual. Individuals with 4 or 5 exposures were collapsed into 1 group. The definition of each exposure was designed to maximize statistical power, while simultaneously ensuring that participants who were sufficiently strongly exposed to each risk factor.

#### TEDS Measures

Bullying was measured using the Multidimensional Peer Victimization Scale at age 12 years.^25^ This measure comprises 16 items, including 4 subscales. Scores on each scale ranged from 0 to 8; we considered participants exposed to bullying if they scored more than 6 on at least 1 subscale. Dependent life events are life events that are associated with an individual’s behavior or circumstances, such as the breakdown of a relationship or experiencing a crime. They were measured using the abbreviated Coddington Life Events Record at age 16 years.^26^ Participants reported whether or not they had experienced 10 dependent life events. We considered them exposed if they experienced at least 3. Cannabis use and tobacco use were both measured using a checklist inquiring about substance use and were assessed by a binary item at age 16 years. If these items were endorsed, participants were considered exposed. Birth weight was reported by the parents at first contact. We defined low birth weight as a birth weight within the lowest 15% of the distribution (<1990 g).

#### CATSS Measures

Bullying was assessed at age 15 years using the Olweus Bully/Victim Questionnaire,^27^ including 16 questions. We considered participants to have been bullied if they reported at least 1 form of bullying, perpetrated mainly by 1 student, and with a duration of 1 to 2 weeks. Dependent life events were measured by a checklist of 29 items at age 18 years, including 13 about dependent life events. We considered participants to be exposed if they endorsed 3 or more dependent life events. Cannabis use and tobacco use were assessed in the same way as in TEDS, at age 18 years. Birth weight was ascertained from the medical birth register. We considered participants to be exposed if their birth weight was in the lowest 15% of the distribution (<2040 g).

#### Psychotic Experiences

In TEDS, psychotic experiences were measured using the Specific Psychotic Experiences Questionnaire (SPEQ)^1^ at age 16 years. The SPEQ was developed from preexisting measures adapted for use in adolescent samples and includes 5 self-reported subscales: paranoia, hallucinations, cognitive disorganization, grandiosity, and anhedonia and a parent-reported negative-symptoms subscale. In CATSS, twins completed the Adolescent Psychotic-Like Symptom Screener (APSS)^28^ at age 18 years, which includes 7 items.

### Statistical Analyses

Before analysis, participants with missing data on the environmental exposure were excluded because they could not be included in moderation twin models. Participants with missing data on the psychotic experience measures were addressed using maximum-likelihood estimation, which is robust to missing data. We tested the phenotypic associations between cumulative exposure to environmental risks and psychotic experiences using linear regression models, implemented as generalized estimating equations to account for related individuals in the samples. Second, we fitted univariate twin models to the APSS in CATSS and environmental exposure variables in both samples. We did not repeat univariate analyses of the SPEQ, as these have been published.^8^

**Figure 1.**
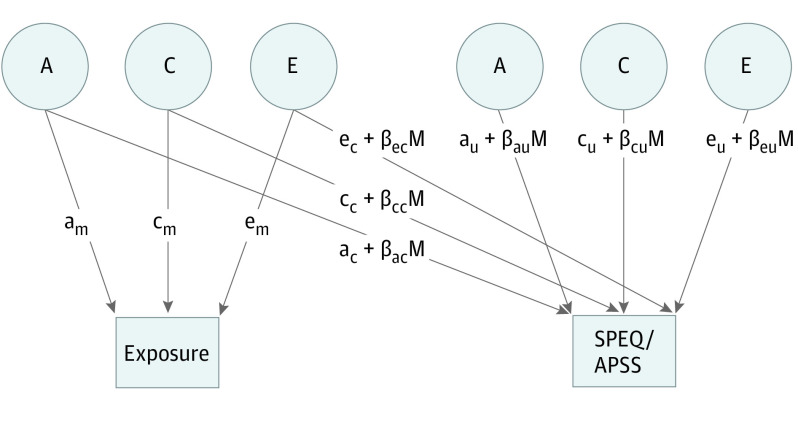
Diagram of a Moderation Model The path diagram is shown for 1 twin only. The Adolescent Psychotic-Like Symptom Screener (APSS) and the Specific Psychotic Experiences Questionnaire (SPEQ) were the 2 measures used to assess psychotic experiences. The variance in the exposure is decomposed into additive genetic (A), shared environmental (C), and nonshared environmental (E) components, which are derived from the path coefficients (labeled a_m_, c_m_, and e_m_ in the path diagram). The paths connecting the exposure with SPEQ/APSS are covariance paths (a_c_, c_c_, and e_c_). The residual variance in SPEQ/APSS is also decomposed into A, C, and E, based on path coefficients a_u_, c_u_, and e_u_. Moderation effects are also estimated for the covariance (β_ac_M, β_cc_M, and β_ec_M) and residual (β_au_M, β_cu_M, and β_eu_M) paths. These moderation effects can be used to calculate the value of each variance component (both the raw variance and the proportion of variance explained) at different levels of the moderator variable.

We tested for gene-environment interaction using moderation models.^29^ These models estimate the genetic and environmental variance in a trait at different levels of a measured environmental exposure (ie, moderator variable). This approach can yield false-positive results in the presence of gene-environment correlation.^30^ Prior studies in TEDS have reported genetic correlations between some of our exposures and psychotic experiences^17,18,19^; therefore, we used the full bivariate moderation model here.^30^ This model accounts for gene-environment correlation and thus the only model that estimates true moderation effects.^30^ Figure 1 shows a diagram of this model. The environmental exposure composite and psychotic experiences were included as manifest variables. The variance in each of these was decomposed into genetic and environmental components, which were then used to calculate the proportion of variance explained by each component. Covariance paths between them are included. The exposure was also included as a moderator; coefficients that correspond to moderation effects for each variance component were estimated. Using these parameters, we calculated the genetic (A) and environmental (C and E) variance associated with each level of exposure. The statistical significance of these effects was then tested by constraining the moderation effects to be equal across exposure groups, first for each variance component separately and then for all components. The statistical significance of these effects was tested using the likelihood-ratio test. We present the results here as the proportions of variance explained by each component for ease of interpretation. Phenotypic analyses were conducted using the drgee package of R (R Foundation).^31^ Twin analyses were conducted using the OpenMx package of R (R Foundation).^32^ Data were analyzed from September 1, 2018, to August 31, 2020.

## Results

Descriptive statistics are in Table 1. A total of 4855 twin pairs (1926 female same-sex pairs, 1397 male same-sex pairs, and 1532 opposite-sex pairs) were included from TEDS, and 6435 twin pairs (2358 female same-sex pairs, 1861 male same-sex pairs, and 2216 opposite-sex pairs) were included from CATSS. Mean age of twins from TEDS was 16.5 years, and the mean age of twins from CATSS was 18.6 years. In TEDS, 43.3% of participants (4209 of 9710) had at least 1 environmental exposure, compared with 52.1% of participants (8932 of 17 136) in CATSS.

**Table 1.** Descriptive Statistics

Variable	No. (%)						
	Overall	MZF	DZF	MZM	DZM	DZOS, female	DZOS, male
**Twins Early Development Study**							
No. of pairs	4855	1028	898	724	673	766	766
SPEQ descriptives, mean (SD)							
Paranoia	12.06 (10.52)	12.24 (10.91)	12.12 (10.29)	10.83 (9.58)	11.62 (10.27)	13.12 (11.02)	12.24 (10.72)
Hallucinations	4.70 (6.09)	4.71 (5.96)	4.77 (6.22)	4.24 (5.99)	4.24 (5.66)	5.60 (6.58)	4.57 (5.99)
Cognitive disorganization	3.96 (2.86)	4.32 (2.86)	4.55 (2.87)	3.18 (2.68)	3.38 (2.76)	4.50 (2.90)	3.51 (2.70)
Grandiosity	5.37 (4.49)	4.94 (4.33)	4.68 (4.15)	5.95 (4.64)	5.99 (4.55)	4.93 (4.36)	6.11 (4.74)
Anhedonia (reversed)	16.71 (7.87)	14.98 (7.38)	15.36 (7.46)	18.69 (7.95)	18.98 (7.85)	14.98 (7.52)	18.55 (7.93)
Negative symptoms	2.82 (3.82)	2.53 (3.57)	2.68 (3.90)	2.75 (3.47)	3.08 (4.05)	2.44 (3.51)	3.57 (4.33)
Exposure frequencies							
Being bullied	1282 (16.5)	258 (15.2)	193 (13.4)	240 (20.7)	200 (18.4)	174 (14.5)	217 (18.1)
Dependent stressful life events	885 (9.7)	171 (8.8)	197 (11.6)	127 (9.4)	112 (8.8)	140 (9.6)	138 (9.7)
Tobacco use	1714 (23.6)	337 (23.2)	324 (24.4)	226 (20.3)	231 (22.1)	314 (26.9)	282 (24.2)
Cannabis use	725 (10.0)	94 (6.5)	113 (8.6)	129 (11.4)	114 (11.0)	123 (10.5)	152 (13.2)
Low birth weight	1422 (15.0)	398 (19.8)	232 (13.3)	235 (16.7)	159 (12.0)	210 (14.0)	188 (12.5)
No. of exposures							
0	5501 (56.7)	1114 (54.2)	1058 (58.9)	784 (54.1)	795 (59.1)	868 (56.7)	882 (57.6)
1	2861 (29.5)	690 (33.6)	505 (28.1)	451 (31.1)	366 (27.2)	440 (28.7)	409 (26.7)
2	958 (9.9)	196 (9.5)	164 (9.1)	146 (10.1)	119 (8.8)	161 (10.5)	172 (11.2)
3	313 (3.2)	48 (2.3)	52 (2.9)	55 (3.8)	52 (3.9)	53 (3.5)	53 (3.5)
4	73 (0.8)	8 (0.4)	15 (0.8)	11 (0.8)	14 (1.0)	10 (0.7)	15 (1.0)
5	4 (0.0)	0 (0.0)	2 (0.1)	1 (0.1)	0 (0.0)	0 (0.0)	1 (0.1)
**Child and Adolescent Twin Study in Sweden**							
No. of pairs	6435	1133	1225	834	1027	1108	1108
APSS descriptives, mean (SD)							
Self-report APSS	1.13 (1.85)	1.22 (1.86)	1.23 (1.85)	0.99 (1.67)	1.02 (1.89)	1.22 (1.95)	0.98 (1.78)
Parent report APSS	0.14 (0.40)	0.14 (0.35)	0.15 (0.43)	0.16 (0.43)	0.16 (0.47)	0.11 (0.32)	0.12 (0.37)
Exposure frequencies							
Being bullied	2450 (22.8)	426 (22.8)	481 (24.0)	297 (21.3)	353 (20.1)	478 (24.9)	415 (23.0)
Dependent life events	3154 (31.9)	602 (32.0)	740 (38.1)	270 (22.7)	366 (26.1)	756 (38.4)	420 (28.2)
Low birth weight	2393 (14.7)	556 (19.7)	435 (14.3)	340 (16.2)	335 (13.0)	431 (14.2)	296 (11.0)
Tobacco use	3373 (25.5)	550 (24.1)	642 (25.7)	373 (21.8)	547 (25.6)	691 (28.8)	570 (26.1)
Cannabis use	687 (5.2)	84 (3.7)	117 (4.7)	98 (5.7)	139 (6.5)	93 (3.9)	156 (7.1)
No. of exposures							
0	8204 (47.9)	1324 (44.7)	1448 (45.1)	1126 (51.2)	1408 (52.1)	1429 (44.5)	1469 (51.6)
1	6337 (37.0)	1148 (38.8)	1236 (38.5)	817 (37.1)	924 (34.2)	1231 (38.4)	981 (34.4)
2	2110 (12.3)	404 (13.6)	418 (13.0)	214 (9.7)	308 (11.4)	438 (13.7)	328 (11.5)
3	441 (2.6)	78 (2.6)	97 (3.0)	43 (2.0)	60 (2.2)	98 (3.1)	65 (2.3)
4+	44 (0.3)	7 (0.2)	13 (0.4)	NA^a^ (0.0)	5 (0.2)	12 (0.4)	6 (0.2)

Abbreviations: APSS, Adolescent Psychotic-Like Symptom Screener; DZF, dizygotic female twins; DZM, dizygotic male twins; DZOS, dizygotic opposite-sex twin pair; MZF, monozygotic female twins; MZM, monozygotic male twins; NA, not applicable; SPEQ, Specific Psychotic Experiences Questionnaire.

^a^ Frequency not shown due to low number.

### Phenotypic Analyses

**Figure 2.**
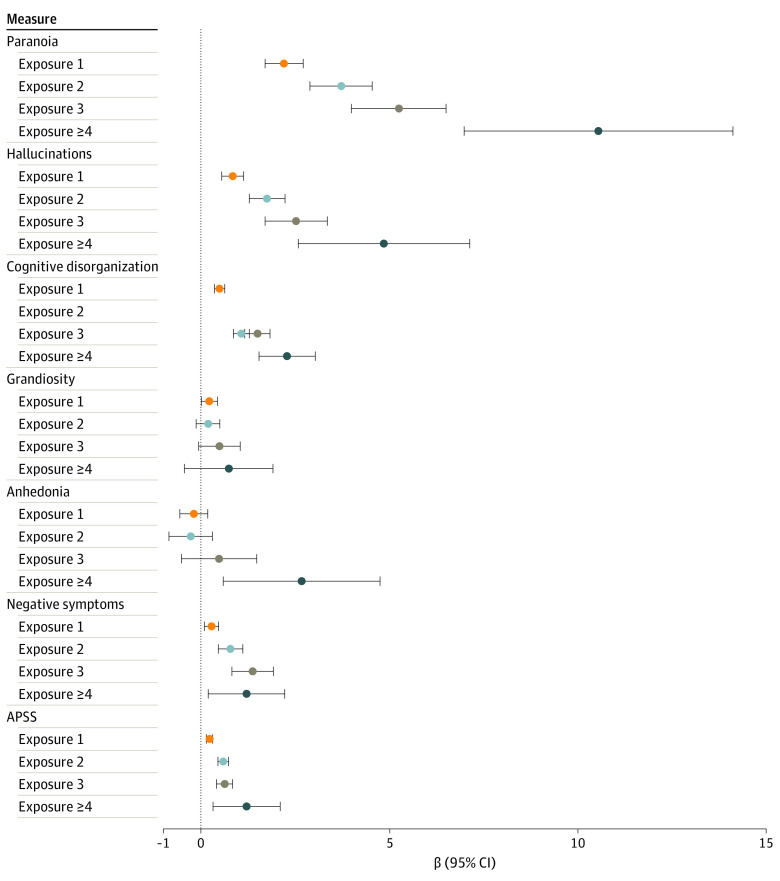
Phenotypic Associations Between Environmental Exposure and Psychotic Experiences All estimates are unstandardized β coefficients calculated from linear regression models of the cumulative exposure variable and each subscale. 95% CIs are based on robust SEs, calculated using generalized estimating equations.

Figure 2 shows regression coefficients from the phenotypic analyses. In TEDS, all levels of exposure were associated with paranoia (1 exposure: β, 2.20; 95% CI, 1.70-2.71; 2 exposures: β, 3.72; 95% CI, 2.89-4.54; 3 exposures: β, 5.25; 95% CI, 3.99-6.50; 4+ exposures: β, 10.54; 95% CI, 6.98-14.11), hallucinations (1 exposure: β, 0.84; 95% CI, 0.55-1.13; 2 exposures: β, 1.75; 95% CI, 1.28-2.23; 3 exposures: β, 2.52; 95% CI, 1.70-3.35; 4+ exposures: β, 4.85; 95% CI, 2.58-7.13), cognitive disorganization (1 exposure: β, 0.49: 95% CI, 0.36-0.63; 2 exposures: β, 1.07; 95% CI, 0.86-1.28; 3 exposures: β, 1.50; 95% CI, 1.16-1.83; 4+ exposures: β, 2.28; 95% CI, 1.54-3.03), and negative symptoms (1 exposure: β, 0.28; 95% CI, 0.09-0.47; 2 exposures: β, 0.78; 95% CI, 0.46-1.11; 3 exposures: β, 1.37; 95% CI, 0.82-1.92; 4+ exposures: β, 1.21; 95% CI, 0.19-2.22). For paranoia, hallucinations, and cognitive disorganization, associations strengthened with increasing exposure, albeit with overlapping CIs. There was a weak association between 1 exposure and grandiosity, whereas 4 or more exposures were associated with anhedonia. In CATSS, all 4 levels of exposure showed a statistically significant association with psychotic experiences, and the association increased with increasing exposure, although CIS overlapped (1 exposure: β, 0.23; 95% CI, 0.14-0.31; 2 exposures: β, 0.59; 95% CI, 0.45-0.73; 3 exposures: β, 0.63; 95% CI, 0.41-0.84; 4+ exposures: β, 1.21; 95% CI, 0.32-2.10).

### Twin Analyses

Univariate results for the APSS and environmental composites are displayed in eTables 2 to 5 in the Supplement. The APSS showed heritability of 23% in boys and 40% in girls. The heritability of the environmental composite was 37% (95% CI, 27%-46%) in TEDS and 24% (range, 19%-29%) in CATSS.

**Figure 3.**
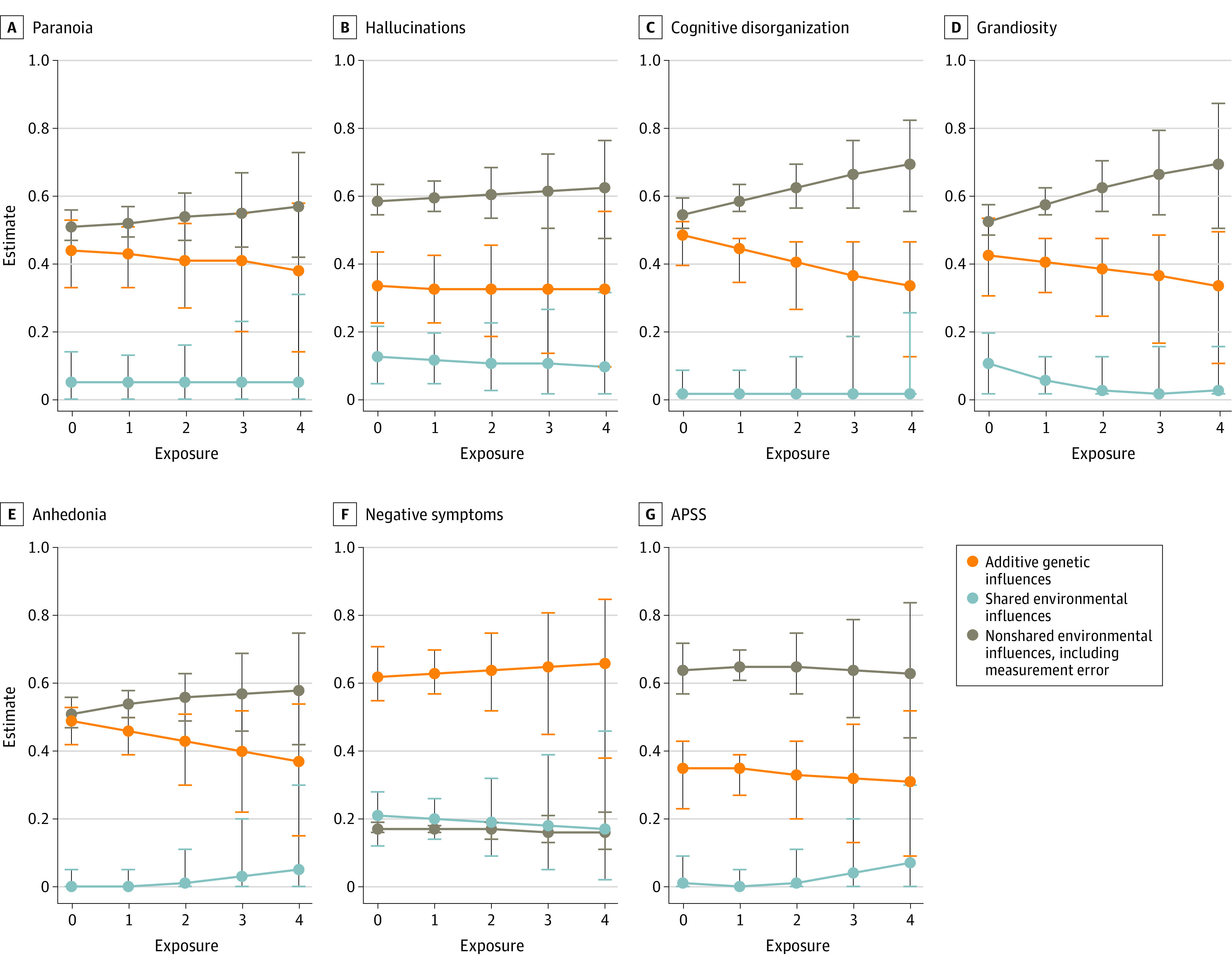
Estimates From the Moderation Models All estimates are given as the proportion of variance in each measure explained by each variance component, separately by exposure group. APSS indicates Adolescent Psychotic-Like Symptom Screener.

Figure 3 shows the estimates from the moderation models (eTables 6 and 7 in the Supplement). Table 2 shows the fit statistics. Statistically significant differences between 2 models indicate statistically significant moderation effects. We observed moderation effects for 5 out of 6 measures (83.3%) in TEDS: paranoia, hallucinations, cognitive disorganization, grandiosity, and anhedonia (of which 4 were statistically significant). Their heritability decreased with increasing environmental exposure. However, fluctuations in the underlying variance components differed across these measures. Paranoia heritability changed from 44% (95% CI, 33%-53%) to 38% (95% CI, 14%-58%) with increasing exposure. Cognitive disorganization heritability changed from 47% (95% CI, 38%-51%) to 32% (95% CI, 11%-45%) across groups, because the total genetic variance decreased, whereas nonshared environmental variance increased. The same was true for grandiosity, where heritability changed from 41% (95% CI, 29%-52%) to 32% (95% CI, 9%-48%), owing to decreasing genetic variance and increasing nonshared environmental variance. For anhedonia, the change in heritability from 49% (95% CI, 42%-53%) to 37% (95% CI, 15%-54%) was attributable to decreasing genetic variance and stable nonshared environmental variance. For hallucinations, heritability was constant, from 32% (95% CI, 21%-42%) in individuals with no exposures to 31% in all 4 exposure groups. Underlying this apparent stability were increases in both genetic and environmental variance. The CATSS sample yielded statistically significant moderation effects, with heritability of psychotic experiences changing from 35% (95% CI, 23%-43%) to 31% (95% CI, 9%-52%) across groups.

**Table 2.** Twin Model Fit Statistics

Model	−2LL^a^	Parameters	*df*	Δχ^2^^b^	Δ*df*^c^	*P* value^d^
**Paranoia**						
Full bivariate	49 291.69	17	19 292	NA	NA	NA
Drop A moderation	49 293.00	16	19 293	1.32	1	.25
Drop C moderation	49 291.70	16	19 293	0.01	1	.91
Drop E moderation	49 292.59	16	19 293	0.90	1	.34
Drop all moderation	49 299.02	14	19 295	7.33	3	.06
**Hallucinations**						
Full bivariate	49 578.99	17	19 302	NA	NA	NA
Drop A moderation	49 579.16	16	19 303	0.16	1	.69
Drop C moderation	49 579.00	16	19 303	0.00	1	.95
Drop E moderation	49 582.82	16	19 303	3.82	1	.05
Drop all moderation	49 588.62	14	19 305	9.63	3	.02
**Cognitive disorganization**						
Full bivariate	49 476.53	17	19 291	NA	NA	NA
Drop A moderation	49 476.87	16	19 292	0.34	1	.56
Drop C moderation	49 476.53	16	19 292	0.00	1	<.99
Drop E moderation	49 488.19	16	19 292	11.66	1	.001
Drop all moderation	49 494.14	14	19 294	17.61	3	.001
**Grandiosity**						
Full bivariate	49 509.94	17	19 246	NA	NA	NA
Drop A moderation	49 510.30	16	19 247	0.37	1	.55
Drop C moderation	49 511.65	16	19 247	1.71	1	.19
Drop E moderation	49 513.53	16	19 247	3.59	1	.06
Drop all moderation	49 518.32	14	19 249	8.38	3	.04
**Anhedonia**						
Full bivariate	49 541.41	17	19 249	NA	NA	NA
Drop A moderation	49 541.43	16	19 250	0.01	1	.92
Drop C moderation	49 541.53	16	19 250	0.11	1	.74
Drop E moderation	49 548.50	16	19 250	7.09	1	.008
Drop all moderation	49 553.57	14	19 252	12.16	3	.007
**Negative symptoms**						
Full bivariate	47 326.21	17	19 327	NA	NA	NA
Drop A moderation	47 326.96	16	19 328	0.75	1	.39
Drop C moderation	47 326.21	16	19 328	0.00	1	.99
Drop E moderation	47 326.65	16	19 328	0.44	1	.51
Drop all moderation	47 332.04	14	19 330	5.83	3	.12
**APSS**						
Full bivariate	90 237.22	17	43 692	NA	NA	NA
Drop A moderation	90 237.77	16	43 693	0.55	1	.46
Drop C moderation	90 237.65	16	43 693	0.43	1	.51
Drop E moderation	90 244.17	16	43 693	6.95	1	.008
Drop all moderation	90 258.61	14	43 695	21.39	3	<.001

Abbreviation: NA, not applicable.

^a^ −2LL = fit statistic, −2 × log likelihood of the data.

^b^ Δχ^2^ = −2LL discrepancy between models, distributed χ^2^.

^c^ The difference in degrees of freedom between the 2 models is equivalent to the difference in number of parameters between 2 models.

^d^ Significant values indicate that a nested model fits statistically significantly more poorly than the model it is being compared to, supporting the statistical significance of the parameter(s) dropped from the model.

## Discussion

In this cohort study, we tested whether the heritability of adolescent psychotic experiences changes with exposure to environmental risks associated with psychotic experiences. Results suggest a gene-by-environment interaction for paranoia, hallucinations, cognitive disorganization, grandiosity, and anhedonia. The findings were replicated in an independent Swedish sample, thus lending robustness to our results. Our study thus suggests differences in heritability of certain psychotic experiences that may be associated with environmental exposures.

Specifically, there was an observed reduction in heritability of psychotic experiences in the presence of environmental exposures. These results are consistent with a bioecological framework, which would predict that more favorable environments would lead to higher heritability.^20^Our results run contrary to a diathesis-stress pathway to psychotic experiences, which would predict that environmental risks trigger a genetic susceptibility to a given disorder and would thus lead to higher heritability of a phenotype in the presence of environmental risks.^20^

It is also important to put our results in the context of prior studies of gene-by-environment interaction in relation to psychotic experiences. One study^33^ reported that the association between environmental risks and psychotic experiences increased among individuals with a family history of psychosis. Although this finding also supports gene-by-environment interaction, the results are somewhat different than what would be expected from our analyses because our analyses suggest that genetic factors were less salient in the presence of environmental exposures. Moreover, other studies^34,35^ have found no evidence of gene-by-environment interaction for psychotic experiences. Methodological differences may underlie these discrepancies. First, family history is not the same as genetic influence because family history includes a combination of genetic and shared environmental factors. Second, we focused on a composite exposure score comprising 5 environmental factors; prior studies have focused on more specific factors, including childhood physical abuse^35^ and trauma.^34^ Third, we focused on 6 specific psychotic experiences here, whereas prior studies used single measures. Finally, it is important to note that prior studies focus specifically on the interaction between genetic risk for psychotic experiences based on family history, by contrast with the current study that used the twin design. In our study, we tested whether heritability differed dependent on exposure to environmental risks. Heritability is a population statistic, and as such, an approach based on calculating heritability is somewhat different from an approach based on using family history as a proxy for individual genetic risk.

On a clinical level, it is first important to clarify that we focused on a young sample, who were aged 16 or 18 years. Many of these individuals will be too young to have been diagnosed with a psychotic disorder, but it is known that psychotic experiences in this age group can lead to severe clinical conditions in some individuals. Nonetheless, clinically it is often noted that many individuals with schizophrenia do not have a family history of schizophrenia. Indeed, although the relative risk for schizophrenia is increased among relatives of individuals diagnosed with schizophrenia, most relatives of individuals with schizophrenia do not develop schizophrenia.^36^ Our results extend these observations to adolescent psychotic experiences and indicate that they may develop in a variety of contexts. Specifically, our results suggest that psychotic experiences may be prevalent in populations with a high degree of exposure to environmental risks associated with psychotic experiences. Indeed, there is substantial conjecture that psychotic experiences can be reached through multiple pathways, such as pathways that are more based on genetic propensity and others that are more down to environmental risks; however, to our knowledge, this is one of the first studies to provide replicable empirical findings on this topic.

The previously mentioned arguments should be tempered, however, for certain types of psychotic experiences. Although we observed that the heritability differed according to environmental exposure for some psychotic experiences, the heritability was more consistent for hallucinations and negative symptoms. This is important from a clinical perspective, given that negative symptoms are thought to be particularly predictive of subsequent mental illness. As such, these results lend further weight to the argument that the etiology of psychotic experiences may differ according to specific subtypes of psychotic experience.^8^

### Strengths and Limitations

Strengths of our study included the use of 2 representative, population-based samples in different countries. In 1 sample, we measured 6 different specific psychotic experiences, enabling us to consider gene-by-environment interactions for different psychotic experiences. There were several limitations, however. Although we employed data from both the UK and Sweden, we still only focused on 2 European countries. Many of our exposures may differ in prevalence across the world, and it would therefore be useful to assess whether similar results emerge in countries with more environmental variability than the UK and Sweden. Our measures of tobacco and cannabis use were more brief than our other measures. Future studies should use more detailed measures. We also created a composite score that involved counting the number of exposures each participant had undergone, which included summing exposures that may have different etiologies or mechanisms underlying their association with psychotic experiences. Further, although our model has controlled for gene environment correlation, we recognize that birth weight is a complex phenotype influenced by parent and child genetics and prenatal environment.

It is further important to be aware that the environmental composite is not identical across TEDS and CATSS. The life events scale, for example, includes items about increasing numbers of quarrels with parents in CATSS, which are not covered in TEDS. Percentile-based cutoffs were used to define low birth weight; birth weight was lower in TEDS than CATSS on average, and therefore, this led to heavier twins being captured in CATSS. Exclusion criteria also differed between samples; individuals with extreme obstetric complications were excluded from TEDS but not CATSS. The fact that we observed similar results between CATSS and TEDS gives us confidence that these differences did not strongly influence our results; however, they should nevertheless be interpreted with these differences in measure in mind. Finally, twins are generally born lighter than singletons. We included birth weight as an exposure here, and hence, individuals with very low birth weight may have been overrepresented in our sample. However, studying birth weight in twins here is unlikely to create any issues for generalizability for 2 reasons. First, our modeling analyses were focused on variance rather than mean differences. Second, twins were compared with twins in the design (not singletons); as such, modest mean differences between singletons and twins in birth weight did not affect the findings.

## Conclusions

To our knowledge, this twin study was the first with results that suggest that environmental factors play a greater role in the etiology of psychotic experiences than genetic factors. For clinicians who may be aware that psychotic disorders are very highly heritable, it is an important message that early manifestations of psychotic experiences during adolescence are not so strongly heritable, especially in the context of higher environmental exposure. Psychotic experiences are likely to manifest in adolescents both with and without a family history of such challenges and further highlight that genetic and environmental risks for psychotic experiences do not operate in isolation from one another.

## Notes

Supplement. **eTable 1.** Description of the Measures **eTable 2.** Assumptions Testing **eTable 3.** Twin Correlations **eTable 4.** Model Fit Statistics **eTable 5.** Univariate Model Estimates **eTable 6.** Moderation Estimates **eTable 7.** Estimates From the Moderation Models
eReferences
