# Supplementary material for: Heritability of Psychotic Experiences in Adolescents and Interaction With Environmental Risk
Source: JAMA Psychiatry. 2022 Aug 3;79(9):889–98. doi: 10.1001/jamapsychiatry.2022.1947 (PMC9350848; doi:10.1001/jamapsychiatry.2022.1947)
Supplement: Supplement. — eTable 1. Description of the Measures eTable 2. Assumptions Testing eTable 3. Twin Correlations eTable 4. Model Fit Statistics eTable 5. Univariate Model Estimates eTable 6. Moderation Estimates eTable 7. Estimates From the Moderation Models eReferences [file jamapsychiatry-e221947-s001.pdf]

Supplementary Online Content

Taylor MJ, Freeman D, Lundström S, Larsson H, Ronald A. Heritability of psychotic experiences in adolescents and interaction with environmental risk. *JAMA Psychiatry*. Published online August 3, 2022. doi:10.1001/jamapsychiatry.2022.1947

eTable 1. Description of the Measures

eTable 2. Assumptions Testing

eTable 3. Twin Correlations

eTable 4. Model Fit Statistics

eTable 5. Univariate Model Estimates

eTable 6. Moderation Estimates

eTable 7. Estimates From the Moderation Models

eReferences

This supplementary material has been provided by the authors to give readers additional information about their work.

© 2022 Taylor MJ et al. *JAMA Psychiatry.*

eTable 1. Description of the Measures

Exposures			
**Exposure**	**Measure in TEDS**	**Measure in CATSS**	**Reference(s) Supporting Inclusion**
Bullying	Multidimensional Peer Victimization Scale at age 12, self-report. This includes 16 items. For each item, participants were asked 'How often during this school year has another pupil done these things to you?' They could answer 'Not at all' (scored as 0), 'once' (1), or 'more than once' (2). The 16 items were divided into four subscales: property destruction, physical abuse, verbal abuse, and social manipulation.	Olweus Bully/Victim Questionnaire at age 15, self-report. This includes 24, although only 16 were included in this study because the remaining eight items cover bullying perpetration. Participants are asked to rate how often particular forms of bullying have happened to them in the past two months, and can answer 'It has not happened to me in the past couple of months', 'only once or twice', '2 or 3 times a week', 'about once a week', or 'several times a week'. They are also asked how many students perpetrated the bullying ('mainly by 1', 'a group of 2-3', 'a group of 4-9', 'a group of 10 or more', or 'by several different students or groups of students') and how long it lasted.	[1,2]
Dependent Stressful Life Events	Coddington Life Events Record at age 16, self-report. An abbreviated version including 20 items was used. Only the ten items that related to dependent life events were included in this study. The checklist includes a list of life events, such as failure of an important exam, hospitalization, and becoming	A list of life events was devised by the CATSS researchers. Of the 29 items completed by the twins at age 18, 13 related to dependent life events, such as death of a close friend or family member, and being the victim of a crime. For each event,	[1,3,4]

© 2022 Taylor MJ et al. *JAMA Psychiatry.*

	involved with drugs. Participants could respond ‘yes’ or ‘no’ to each item. We did not account for how stressful each event was rated as.	participants answered either ‘it has happened’ or ‘it has not happened’.	
Tobacco Use	A questionnaire about substance and drug use was sent to the TEDS participants. For each drug or substance, they were asked to report whether they had ever tried it or not. One item concerned tobacco.	As for TEDS, a questionnaire was devised and included an item about whether participants had ever tried tobacco or not.	[5,6]
Cannabis Use	The same questionnaire used to assess tobacco use also included an item about cannabis use.	The same questionnaire used to assess tobacco use also included an item about cannabis use.	[7–9]
Birth Weight	When the parents of the twins in TEDS were first contacted, they were asked to complete a questionnaire which provided background and demographic information, as well as some items about perinatal factors. Parents reported the twins’ birth weights in grams.	Birth weight was derived by linking CATSS with the Medical Birth Register, which records all births in Sweden since 1973. Information about birth weight is recorded (in grams) when a new birth is registered.	[5]

Psychotic Experiences

Measure	Rater	N Items	Description of Items
SPEQ Paranoia[10]	Self-Report	15	Participants are asked ‘Have you often thought…?’ and then asked to respond to the items. The items enquire about feeling like others are deliberately trying to cause participants’ harm, feeling as though being spied on or watched, and receiving coded messages from the television or internet. Responses are on a 0-5 Likert scale.
SPEQ Hallucinations[11]	Self-Report	9	Participants are asked ‘How often do you…’?’ and then provided with items about different hallucinations. These include hearing, seeing, or smelling things that others cannot, as well as experiencing unusual sensations that cannot be explained. Responses are on a 0-5 Likert scale.
SPEQ Cognitive Disorganisation[12]	Self-Report	11	Participants are asked to answer the questions based on their feelings in the past month. These include daydreaming, confusion

© 2022 Taylor MJ et al. *JAMA Psychiatry.*

			if too much happens at once, and difficulty in getting started with tasks. Each question can be answered ‘yes’ or ‘no’.
SPEQ Grandiosity[12,13]	Self-Report	8	Participants are asked to answer the questions while considering the last month. The questions cover belief in a special mission, having many great ideas, and being more unique than everyone else. Questions are answered on a 0-3 Likert scale.
SPEQ Anhedonia[14]	Self-Report	10	Participants completed a measure of hedonia, however the items are all reversed so that the items cover *anhedonia*. Participants are asked to answer the questions in relation to the previous month. The items relate heavily to looking forward to things, such as eating at a restaurant. Questions are answered on a 0-5 Likert scale.
SPEQ Negative Symptoms[15]	Parent Report	10	Parents were asked to rate how strongly they agree or disagree with a number of statements in relation to the twins, with no reference to a particular period of time. Items include, among others, lack of emotion, lacking energy or motivation, and having few interests. Questions are answered on a 0-3 Likert scale.
APSS (CATSS)	Self-Report	7	Participants were presented with a number of questions, and asked how often they had experienced various psychotic experiences. These included feeling as though they are being spied on, being sent messages, being conspired against by others, and having visual hallucinations. Questions were not asked with reference to a particular period of time. They were answered on a 0-3 Likert scale.

Note that the Exposures were selected on the basis of prior research showing that they are associated with psychotic experiences; the references given are example studies showing that these exposures are linked with psychotic experiences

© 2022 Taylor MJ et al. *JAMA Psychiatry.*

Univariate Twin Models

eTable 2. Assumptions Testing

Model	-2LL	Parameters	df	Comparison Model	$\Delta\chi^2$	Δdf	p
APSS							
Fully Saturated	22713.67	25	8066	-----	-----	-----	-----
Submodel 1	22715.60	21	8070	Fully Saturated	1.93	4	0.748
Submodel 2	22716.62	17	8074	Fully Saturated	2.95	8	0.938
Submodel 3	22716.95	15	8076	Fully Saturated	3.27	10	0.974
Submodel 4	22718.15	13	8078	Fully Saturated	4.48	12	0.973
Environmental Composite, TEDS							
Fully Saturated	19372.91	45	9665	-----	-----	-----	-----
Submodel 1	19386.38	29	9681	Fully Saturated	13.47	16	0.638
Submodel 2	19410.24	21	9689	Fully Saturated	37.33	24	0.041
Environmental Composite, CATSS							
Fully Saturated	54748.31	45	35573	-----	-----	-----	-----
Submodel 1	54762.46	29	35589	Fully Saturated	14.15	16	0.587
Submodel 2	54785.79	21	35597	Fully Saturated	37.48	24	0.039

For the APSS, the assumptions are: equal means across twin order (submodel 1), equal variances across twin order (submodel 2), equal means across zygosity (submodel 3), and equal variances across zygosity (submodel 4). For the environmental composites, the assumptions are: equal thresholds across twin order (submodel 1) and equal thresholds across zygosity (submodel 2).

© 2022 Taylor MJ et al. *JAMA Psychiatry.*

eTable 3. Twin Correlations

	MZF	DZF	MZM	DZM	DZOS
APSS	0.44 (0.38-0.50)	0.23 (0.16-0.30)	0.26 (0.16-0.35)	0.12 (0.03-0.21)	0.12 (0.06-0.18)
Environmental Exposure, TEDS	0.68 (0.63-0.73)	0.50 (0.43-0.56)	0.69 (0.64-0.74)	0.54 (0.47-0.61)	0.51 (0.46-0.56)
Environmental Exposure, CATSS	0.70 (0.68-0.73)	0.63 (0.60-0.66)	0.74 (0.71-0.77)	0.66 (0.63-0.69)	0.56 (0.54-0.59)

MZF: monozygotic female twins; DZF: dizygotic female twins; MZM: monozygotic male twins; DZM: dizygotic male twins; DZOS: dizygotic opposite-sex twins; APSS: Adolescent Psychotic-Like Symptom Screener

© 2022 Taylor MJ et al. *JAMA Psychiatry.*

eTable 4. Model Fit Statistics

Model	-2LL	Parameters	df	Comparison Model	Δχ^2	Δdf	p
APSS, Self-Report							
Fully Saturated	22713.67	25	8066	-----	-----	-----	-----
ACE	22728.88	9	8082	Fully Saturated	15.21	16	0.509
ADE	22728.90	9	8082	Fully Saturated	15.23	16	0.508
Quan	22729.08	8	8083	ACE	0.20	1	0.656
Hom	22745.77	5	8086	Quan	16.69	3	0.001
AE	22731.13	6	8085	Quan	2.05	2	0.359
CE	22764.91	6	8085	Quan	35.83	2	<0.001
E	22960.05	4	8087	Quan	230.97	4	<0.001
Fully Saturated	19372.91	45	9665	-----	-----	-----	-----
ACE	19420.44	14	9702	Fully Saturated	47.53	37	0.115
Hom	19421.07	11	9705	ACE	0.63	3	0.89
AE	19484.09	10	9706	Hom	63.02	1	<0.001
CE	19473.48	10	9706	Hom	52.42	1	<0.001
E	20565.4	9	9707	Hom	1144.33	2	<0.001
Fully Saturated	54748.31	45	35573	-----	-----	-----	-----
ACE	54801.18	14	35610	Fully Saturated	52.87	37	0.044
Hom	54837.58	11	35613	ACE	36.39	3	<0.001
AE	55303.92	10	35614	Hom	466.34	1	<0.001
CE	54915.94	10	35614	Hom	78.37	1	<0.001
E	59112.99	9	35615	Hom	4275.41	2	<0.001

*-2LL: fit statistic, -2*log-likelihood of the data; df: degrees of freedom; Δχ^2: -2LL discrepancy between models, distributed χ^2; Δdf: difference in degrees of freedom between two models, equivalent to the difference in number of parameters between two models; p: p-value for the comparison*

© 2022 Taylor MJ et al. *JAMA Psychiatry.*

between two models; significant values indicate that a nested model fits statistically significantly more poorly than the model it is being compared to, supporting the statistical significance of the parameter(s) dropped from the model.
For the APSS, the initial ACE or ADE model was fitted with qualitative sex limitation, then with just quantitative sex limitation ('Quan'). All sex differences were then dropped ('Hom'). Due to low power, only quantitative sex differences were tested for the environmental composites.

eTable 5. Univariate Model Estimates

		A	C	E
APSS	Female	0.40 (0.24-0.48)	0.03 (0.00-0.17)	0.57 (0.52-0.63)
	Male	0.23 (0.01-0.33)	0.02 (0.00-0.19)	0.74 (0.66-0.84)
Exposure Variable, TEDS		0.37 (0.27-0.46)	0.33 (0.25-0.40)	0.31 (0.28-0.32)
Exposure Variable, CATSS		0.24 (0.19-0.29)	0.48 (0.44-0.52)	0.27 (0.26-0.30)

A: additive genetic influences; C: nonadditive genetic influences; E: nonshared environmental influences; APSS: Adolescent Psychotic-Like Symptoms Screener

© 2022 Taylor MJ et al. *JAMA Psychiatry.*

Moderation Results

eTable 6. Moderation Estimates

Measure	A Moderation	C Moderation	E Moderation
Paranoia	-0.03 (-0.08-0.02)	0.01 (-0.09-0.11)	-0.01 (-0.04-0.01)
Hallucinations	0.02 (-0.06-0.08)	0.00 (-0.09-0.10)	0.03 (0.00-0.06)
Cognitive Disorganization	-0.02 (-0.07-0.03)	0.00 (-0.13-0.13)	0.05 (0.02-0.08)
Grandiosity	-0.01 (-0.09-0.04)	0.09 (-0.06-0.06)	0.03 (0.00-0.06)
Anhedonia	0.00 (-0.06-0.05)	-0.07 (-0.18-0.18)	0.04 (0.01-0.07)
Negative Symptoms	0.02 (-0.03-0.07)	0.02 (-0.08-0.10)	0.01 (-0.01-0.03)
APSS in CATSS	0.03 (-0.05-0.11)	0.10 (-0.20-0.20)	0.05 (0.01-0.10)

eTable 7. Estimates From the Moderation Models

	A Variance	C Variance	E Variance	Total Variance	Proportion A	Proportion C	Proportion E
Paranoia							
0	0.44 (0.32-0.53)	0.05 (0.00-0.13)	0.50 (0.46-0.54)	0.99 (0.92-1.04)	0.44 (0.33-0.53)	0.05 (0.00-0.14)	0.51 (0.47-0.56)
1	0.40 (0.30-0.49)	0.05 (0.00-0.12)	0.48 (0.45-0.52)	0.93 (0.87-0.97)	0.43 (0.33-0.51)	0.05 (0.00-0.13)	0.52 (0.48-0.57)
2	0.36 (0.23-0.47)	0.05 (0.00-0.14)	0.47 (0.41-0.53)	0.87 (0.79-0.94)	0.41 (0.27-0.52)	0.05 (0.00-0.16)	0.54 (0.47-0.61)
3	0.32 (0.17-0.48)	0.04 (0.00-0.19)	0.45 (0.37-0.54)	0.82 (0.70-0.91)	0.39 (0.20-0.55)	0.05 (0.00-0.23)	0.55 (0.45-0.67)
4	0.29 (0.11-0.50)	0.04 (0.00-0.26)	0.43 (0.33-0.56)	0.76 (0.62-0.90)	0.38 (0.14-0.58)	0.05 (0.00-0.31)	0.57 (0.42-0.73)
Hallucinations							
0	0.30 (0.19-0.40)	0.10 (0.02-0.19)	0.54 (0.50-0.58)	0.94 (0.89-0.98)	0.32 (0.21-0.42)	0.11 (0.03-0.20)	0.57 (0.53-0.62)
1	0.31 (0.21-0.41)	0.10 (0.03-0.18)	0.58 (0.54-0.63)	1.00 (0.96-1.03)	0.31 (0.21-0.41)	0.10 (0.03-0.18)	0.58 (0.54-0.63)
2	0.33 (0.18-0.48)	0.10 (0.01-0.22)	0.63 (0.55-0.71)	1.06 (0.98-1.12)	0.31 (0.17-0.44)	0.09 (0.01-0.21)	0.59 (0.52-0.67)

© 2022 Taylor MJ et al. *JAMA Psychiatry.*

3	0.35 (0.14-0.58)	0.10 (0.00-0.29)	0.67 (0.56-0.80)	1.12 (1.01-1.23)	0.31 (0.12-0.49)	0.09 (0.00-0.25)	0.60 (0.49-0.71)
4	0.37 (0.10-0.70)	0.10 (0.00-0.39)	0.72 (0.56-0.90)	1.18 (1.03-1.35)	0.31 (0.08-0.54)	0.08 (0.00-0.30)	0.61 (0.46-0.75)
Cognitive Disorganisation							
0	0.44 (0.35-0.48)	0.00 (0.00-0.06)	0.50 (0.46-0.54)	0.93 (0.89-0.97)	0.47 (0.38-0.51)	0.00 (0.00-0.07)	0.53 (0.49-0.58)
1	0.42 (0.32-0.47)	0.00 (0.00-0.07)	0.57 (0.53-0.61)	0.99 (0.95-1.02)	0.43 (0.33-0.46)	0.00 (0.00-0.07)	0.57 (0.54-0.62)
2	0.41 (0.26-0.49)	0.00 (0.00-0.12)	0.65 (0.58-0.72)	1.05 (0.99-1.11)	0.39 (0.25-0.45)	0.00 (0.00-0.11)	0.61 (0.55-0.68)
3	0.39 (0.19-0.52)	0.00 (0.00-0.20)	0.73 (0.61-0.86)	1.12 (1.02-1.22)	0.35 (0.17-0.45)	0.00 (0.00-0.17)	0.65 (0.55-0.75)
4	0.38 (0.14-0.56)	0.00 (0.00-0.32)	0.81 (0.65-1.01)	1.19 (1.06-1.38)	0.32 (0.11-0.45)	0.00 (0.00-0.24)	0.68 (0.54-0.81)
Grandiosity							
0	0.41 (0.30-0.52)	0.09 (0.00-0.18)	0.51 (0.47-0.56)	1.01 (0.95-1.05)	0.41 (0.29-0.52)	0.09 (0.00-0.18)	0.51 (0.47-0.56)
1	0.39 (0.30-0.46)	0.04 (0.00-0.11)	0.55 (0.52-0.59)	0.98 (0.93-1.02)	0.39 (0.30-0.46)	0.04 (0.00-0.11)	0.56 (0.53-0.61)
2	0.36 (0.22-0.46)	0.01 (0.00-0.11)	0.60 (0.53-0.67)	0.97 (0.90-1.03)	0.37 (0.23-0.46)	0.01 (0.00-0.11)	0.61 (0.54-0.69)
3	0.34 (0.15-0.49)	0.00 (0.00-0.13)	0.64 (0.53-0.77)	0.99 (0.87-1.09)	0.35 (0.15-0.47)	0.00 (0.00-0.14)	0.65 (0.53-0.78)
4	0.32 (0.09-0.53)	0.01 (0.00-0.16)	0.69 (0.53-0.88)	1.02 (0.85-1.21)	0.32 (0.09-0.48)	0.01 (0.00-0.14)	0.68 (0.49-0.86)
Anhedonia							
0	0.47 (0.40-0.52)	0.00 (0.00-0.05)	0.49 (0.45-0.53)	0.96 (0.91-0.99)	0.49 (0.42-0.53)	0.00 (0.00-0.05)	0.51 (0.47-0.56)
1	0.47 (0.39-0.52)	0.00 (0.00-0.05)	0.55 (0.51-0.59)	1.02 (0.97-1.06)	0.46 (0.39-0.50)	0.00 (0.00-0.05)	0.54 (0.50-0.58)
2	0.47 (0.33-0.57)	0.01 (0.00-0.13)	0.61 (0.54-0.69)	1.10 (1.01-1.17)	0.43 (0.30-0.51)	0.01 (0.00-0.11)	0.56 (0.49-0.63)
3	0.48 (0.27-0.64)	0.03 (0.00-0.25)	0.68 (0.56-0.82)	1.19 (1.03-1.32)	0.40 (0.22-0.52)	0.03 (0.00-0.20)	0.57 (0.46-0.69)

© 2022 Taylor MJ et al. *JAMA Psychiatry.*

4	0.48 (0.21-0.72)	0.06 (0.00-0.44)	0.75 (0.58-0.95)	1.29 (1.06-1.55)	0.37 (0.15-0.54)	0.05 (0.00-0.30)	0.58 (0.42-0.75)
Negative Symptoms							
0	0.59 (0.52-0.67)	0.20 (0.11-0.27)	0.16 (0.15-0.18)	0.96 (0.92-1.00)	0.62 (0.55-0.71)	0.21 (0.12-0.28)	0.17 (0.16-0.19)
1	0.63 (0.57-0.70)	0.20 (0.14-0.26)	0.17 (0.16-0.18)	1.00 (0.96-1.04)	0.63 (0.57-0.70)	0.20 (0.14-0.26)	0.17 (0.16-0.18)
2	0.68 (0.55-0.80)	0.20 (0.09-0.34)	0.18 (0.15-0.20)	1.05 (0.98-1.13)	0.64 (0.52-0.75)	0.19 (0.09-0.32)	0.17 (0.14-0.19)
3	0.72 (0.51-0.93)	0.20 (0.05-0.45)	0.18 (0.14-0.23)	1.10 (0.99-1.23)	0.65 (0.45-0.81)	0.18 (0.05-0.39)	0.16 (0.13-0.21)
4	0.77 (0.48-1.07)	0.20 (0.02-0.59)	0.19 (0.14-0.25)	1.16 (1.00-1.35)	0.66 (0.38-0.85)	0.17 (0.02-0.46)	0.16 (0.11-0.22)
APSS							
0	0.31 (0.20-0.38)	0.01 (0.00-0.08)	0.56 (0.51-0.63)	0.88 (0.82-0.92)	0.35 (0.23-0.43)	0.01 (0.00-0.09)	0.64 (0.57-0.72)
1	0.34 (0.26-0.40)	0.00 (0.00-0.05)	0.65 (0.60-0.70)	0.99 (0.95-1.04)	0.35 (0.27-0.39)	0.00 (0.00-0.05)	0.65 (0.61-0.70)
2	0.38 (0.22-0.50)	0.01 (0.00-0.13)	0.74 (0.64-0.85)	1.14 (1.05-1.20)	0.33 (0.20-0.43)	0.01 (0.00-0.11)	0.65 (0.57-0.75)
3	0.42 (0.18-0.64)	0.05 (0.00-0.28)	0.84 (0.67-1.03)	1.31 (1.14-1.46)	0.32 (0.13-0.48)	0.04 (0.00-0.20)	0.64 (0.50-0.79)
4	0.46 (0.14-0.82)	0.10 (0.00-0.52)	0.94 (0.70-1.23)	1.50 (1.23-1.81)	0.31 (0.09-0.52)	0.07 (0.00-0.30)	0.63 (0.44-0.84)

A variance, C variance, and E variance represent the decomposition of the total variance into three components. The proportions are the proportion of total variance explained by each component, and were calculated by dividing each component by the total variance.

© 2022 Taylor MJ et al. *JAMA Psychiatry.*

eReferences

1. Arseneault L, Cannon M, Fisher HL, Polanczyk G, Moffitt TE, Caspi A. Childhood trauma and children's emerging psychotic symptoms. *Am J Psychiatry*. 2011;168(1):65-72. http://search.ebscohost.com/login.aspx?direct=true&db=psyh&AN=2011-02156-012&lang=de&site=ehost-live%0Aorcid: 0000-0002-8589-6760%0Aorcid: 0000-0003-4174-2126
2. Shakoor S, Mcguire P, Cardno AG, Freeman D, Plomin R, Ronald A. A Shared Genetic Propensity Underlies Experiences of Bullying Victimization in Late Childhood and Self-Rated Paranoid Thinking in Adolescence. *Schizophr Bull*. 2015;41(3):754-763. doi:10.1093/schbul/sbu142
3. Kelleher I, Keeley H, Corcoran P, et al. Childhood trauma and psychosis in a prospective cohort study: Cause, effect, and directionality. *Am J Psychiatry*. 2013;170(7):734-741. doi:10.1176/appi.ajp.2012.12091169
4. Shakoor S, Zavos HMS, Haworth CMA, et al. Association between stressful life events and psychotic experiences in adolescence: Evidence for gene–environment correlations. *Br J Psychiatry*. 2016;208(6):532-538. doi:10.1192/bjp.bp.114.159079
5. Fusar-Poli P, Tantardini M, De Simone S, et al. Deconstructing vulnerability for psychosis: Meta-analysis of environmental risk factors for psychosis in subjects at ultra high-risk. *Eur Psychiatry*. 2017;40:65-75. doi:10.1016/j.eurpsy.2016.09.003
6. Barkhuizen W, Taylor MJ, Freeman D, Ronald A. A Twin Study on the Association Between Psychotic Experiences and Tobacco Use During Adolescence. *J Am Acad Child Adolesc Psychiatry*. 2019;58(2):267-276.e8. doi:10.1016/j.jaac.2018.06.037
7. Morgan C, Reininghaus U, Reichenberg A, Frissa S, Hotopf M, Hatch SL. Adversity, cannabis use and psychotic experiences: Evidence of cumulative and synergistic effects. *Br J Psychiatry*. 2014;204(5):346-353. doi:10.1192/bjp.bp.113.134452
8. Harley M, Kelleher I, Clarke M, et al. Cannabis use and childhood trauma interact additively to increase the risk of psychotic symptoms in adolescence. *Psychol Med*. 2010;40(10):1627-1634. doi:10.1017/S0033291709991966
9. Shakoor S, Zavos HMS, McGuire P, Cardno AG, Freeman D, Ronald A. Psychotic experiences are linked to cannabis use in adolescents in the community because of common underlying environmental risk factors. *Psychiatry Res*. 2015;227(2-3):144-151. doi:10.1016/J.PSYCHRES.2015.03.041
10. Freeman D, Garety PA, Bebbington PE, et al. Psychological investigation of the structure of paranoia in a non-clinical population. *Br J Psychiatry*. 2005;186(MAY):427-435. doi:10.1192/BJP.186.5.427
11. Bell V, Halligan PW, Ellis HD. The Cardiff Anomalous Perceptions Scale (CAPS): A New Validated Measure of Anomalous Perceptual Experience. doi:10.1093/schbul/sbj014
12. Mason O, Linney Y, Claridge G. Short scales for measuring schizotypy. *Schizophr Res*. 2005;78(2-3):293-296. doi:10.1016/J.SCHRES.2005.06.020
13. Peters E, Joseph S, Day S, Qarety P. *Measuring Delusional Ideation: The 21-Item Peters et AL Delusions Inventory (PDI)*. https://academic.oup.com/schizophreniabulletin/article/30/4/1005/1930847
14. Gard DE, Gard MG, Kring AM, John OP. Anticipatory and consummatory components of the experience of pleasure: A scale development study. *J Res Pers*. 2006;40(6):1086-1102. doi:10.1016/J.JRP.2005.11.001
15. Andreasen N. *The Scale for the Assessment of Negative Symptoms (SANS)*. Iowa: University of Iowa, 1984.

© 2022 Taylor MJ et al. *JAMA Psychiatry.*
